# Integrated analysis of expression, prognostic value and immune infiltration of GSDMs in hepatocellular carcinoma

**DOI:** 10.18632/aging.203669

**Published:** 2021-11-03

**Authors:** Kuan Hu, Zhijie Xu, Lei Yao, Yuanliang Yan, Lei Zhou, Juanni Li

**Affiliations:** 1 Department of Hepatobiliary Surgery, Xiangya Hospital, Central South University, Changsha 410008, Hunan, China; 2 Department of Pathology, Xiangya Hospital, Central South University, Changsha 410008, Hunan, China; 3 Department of Pharmacy, Xiangya Hospital, Central South University, Changsha 410008, Hunan, China; 4 National Clinical Research Center for Geriatric Disorders, Xiangya Hospital, Central South University, Changsha 410008, Hunan, China; 5 Department of Anesthesiology, Third Xiangya Hospital of Central South University, Changsha 410008, Hunan, China

**Keywords:** GSDM family, hepatocellular carcinoma, expression profiles, prognosis, immune infiltration

## Abstract

Six Gasdermins (GSDM) family members participate in various biological processes especially pyroptosis, as well as in the initiation and development of many types of cancer. However, the systematic analysis of the GSDM family in hepatocellular carcinoma (HCC) is lacking. In this study, several bioinformatics databases were recruited to analyze the roles of the GSDMs in differential expression, prognostic correlation, functional enrichment exploration, immune modulation, genetic alterations, and methylated modification in patients with HCC. Consequently, the mRNA expression of all the six GSDMs was accordantly increased in HCC, while only the protein expressions of GSDMB, GSDMD, and GSDME were apparently increased in HCC tissue. The expression of all the GSDMs (except GSDMA) was significantly higher in tumor stage 1–3 subgroups, compared with that in normal subgroups. Higher GSDME expression was significantly associated with shorter overall survival (OS) and disease specific survival (DSS) in patients with HCC. GSDMD had the highest genetic alteration rate among the GSDMs. The three signal pathways which were most likely related to GSDMs-associated molecules were the cell adhesion, growth regulation, and hormone metabolic process. The majority of GSDMs members were positively correlated with the infiltration of B cells, neutrophils, and dendritic cells, however negatively correlated with macrophage. All of the six GSDM members showed remarkably decreased methylation levels in HCC tissues. In conclusion, the GSDM family (especially GSDME) had the potential to become essential biomarkers to better improve the diagnosis and prognosis of HCC, as well as provided insight for the development of therapeutic targets.

## INTRODUCTION

Liver cancer is the leading cause of cancer-related deaths globally, with the foremost morbidity and incidence among all types of cancer. Hepatocellular carcinoma (HCC) as the most common subtype in liver cancer, is characterized by the insidious onset, high malignancy, and awfully poor clinical outcomes [1, 2]. It is reported that merely 30% of patients with HCC are at the early stage which is suitable for radical surgery. Moreover, the effectiveness of chemotherapeutic drugs, targeted drugs, and immunotherapeutic agents for advanced HCC remains limited [3, 4]. Hence exploring novel biomarkers and molecular targets is of great value for the development of HCC therapeutic strategy.

Gasdermins (GSDM) family members are six pore-forming effector proteins that were identified very recently, including GSDMA, GSDMB, GSDMC, GSDMD, GSDME (DFNA5), and PJVK (Pejvakin, DFNB59). All the six GSDMs share a highly conserved pore-forming domain which is responsible for the membrane permeabilization and cell pyroptosis [5–7]. Pyroptosis is a novel form of lytic programmed cell death with the characterization of pro-inflammation that is broadly involved in a variety of biological processes such as human development and immune response [8–10]. Besides, emerging researches have revealed the strong correlation between the dysregulation of GSDMs and the initiation and development of a various type of cancer [11]. For instance, a study demonstrated that GSDMB induced by lymphocyte-derived granzyme A (GZMA) could trigger the pore-forming activity and pyroptosis, thus promoting anti-tumor immunity [12]. In addition, rs8067378 polymorphism conspicuously increased the expression of GSDMB and the initiation of cervical squamous cell carcinomas [13]. Furthermore, GSDMC was proved to act as an oncogene in colorectal cancer based on the observation that silence of GSDMC remarkably decreased the proliferation of colorectal cancer cells [14]. GSDME was shown to play the role of a tumor suppressor through the mechanisms of pyroptosis activation and increased anti-tumor immunity [15]. Nevertheless, the roles of the six GSDM family members in HCC have not been comprehensively studied before.

Here, we systematically analyzed the GSDM family members in the aspects of the expression profiles, prognostic value, status of genetic alteration, functional enrichment analysis, immune cell infiltration, and methylation status by the usage of multiple public databases. We believed this study may extend the recognition of the role of GSDMs in HCC and provide trains of thought and clues for further mechanistic investigation.

## RESULTS

### Aberrantly increased expression of GSDM family members in patients with HCC

**Figure 1:**
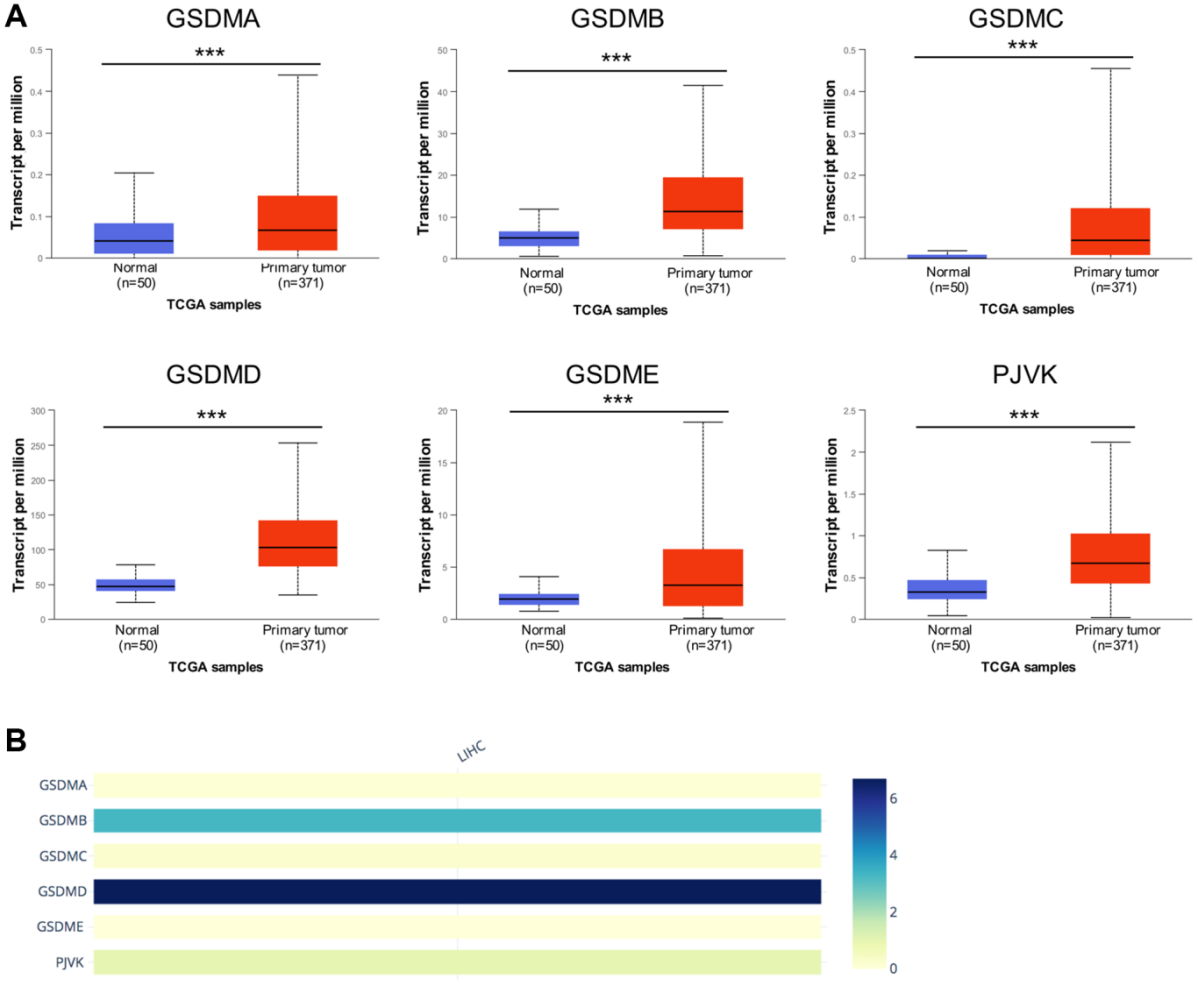
**The mRNA expression levels of six GSDM family members in HCC.** (**A**) GSDMs mRNA expression profiles were collected from the UALCAN database. (**B**) The relative mRNA expression level of individual GSDM family members in HCC. ^***^p < 0.001.

UALCAN databases were enrolled to detect the mRNA expression of the GSDM family members in HCC. Notably, the mRNA expression of all the six GSDMs was accordantly higher in HCC tumor tissue compared with normal tissue (Figure 1A). Then the immunohistochemistry (IHC) staining which represented the protein expression of GSDMs were obtained through the Human Protein Atlas database (THPA) (Supplementary Figure 1), showing that the protein expressions of GSDMB, GSDMD, and GSDME were higher in HCC tissue compared with normal tissue, which is in accordance with the tendency of their mRNA expression. While the protein expressions of GSDMA and GSDMC could not be detected in IHC staining. A probable reason for this inconsistency maybe because of the low value of transcript per million (TPM) of GSDMA and GSDMC, which made their protein expression abundances relatively hard to be detected by IHC. No IHC data of PJVK was retrieved from the THPA database. In addition, the relative expression level within the GSDM family members are ranked as below from high to low: GSDMD, GSDMB, PJVK, GSDMA, GSDMC, and GSDME (Figure 1B).

### The association between GSDMs and the clinicopathological parameters in patients with HCC

**Figure 2:**
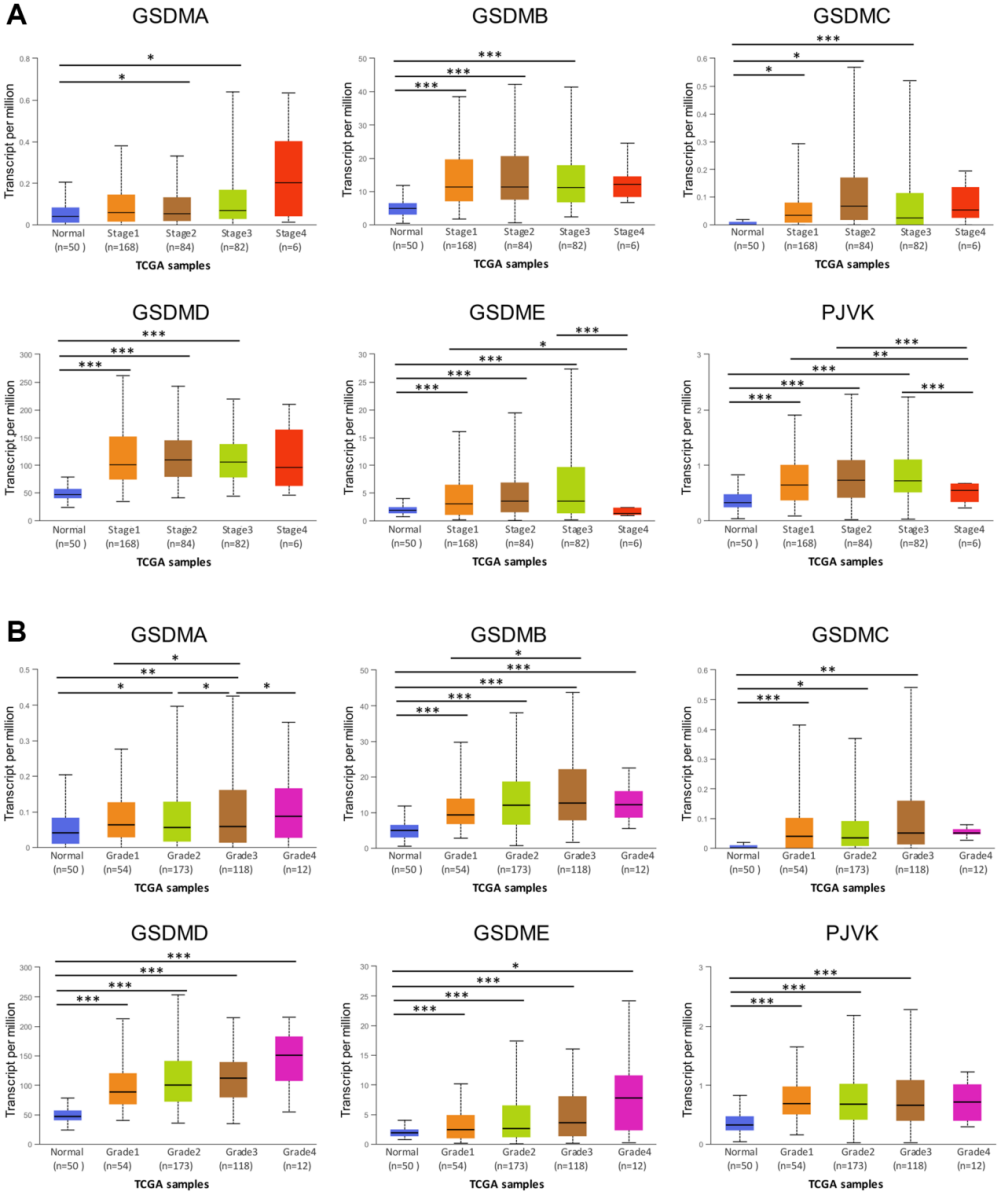
**Association of GSDMs mRNA expression levels with clinical pathology from UALCAN.** (**A**) Relationships between GSDMs transcript levels and individual cancer stages of HCC. (**B**) Relationships between GSDMs transcript levels and tumor grades of HCC. ^*^p < 0.05, ^**^p < 0.01, ^***^p < 0.001.

The tumor stage and tumor grades, the two important clinicopathological parameters were further analyzed in the context of GSDMs expression. The expression of all the six GSDMs (except GSDMA) was significantly higher in tumor stage 1–3 subgroups, compared with that in normal subgroups (Figure 2A). However, there was no difference when it came to tumor stage 4. Besides, the GSDME and PJVK expressions in tumor stage 4 were contrarily decreased compared to tumor stages 1 and 3. These results suggested that there might exist some mechanisms in advanced stages of HCC which hampered the increased expression of GSDMs. As for the tumor grade, GSDMD and GSDME expression increased gradually and significantly from grade 1 to grade 4. For the case of GSDMA, GSDMB, GSDMC, and PJVK, this trend of increased expression still existed but lack the statistic difference (Figure 2B). These data indicated that tumor cell differentiation may to some extent reflected the GSDMs.

### The prognostic role of GSDMs in patients with HCC

**Figure 3:**
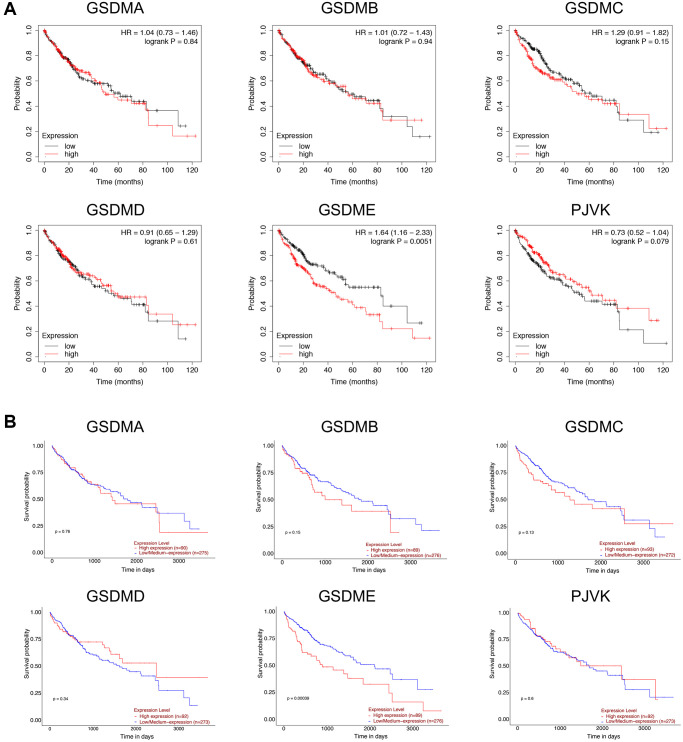
**Prognostic value of GSDMs mRNA expression levels in HCC.** (**A**) Relationships between GSDMs transcript levels and overall survival (OS) of HCC patients were conducted using Kaplan-Meier plotter. (**B**) Relationships between GSDMs transcript levels and overall survival (OS) of HCC patients were analyzed through the UALCAN database.

Overall survival (OS) was used as the primary endpoint to assess the prognostic role of each GSDM family member, data returned from Kaplan–Meier plotter and UALCAN databases gave consistent results, namely only high GSDME expression was significantly associated with shorter OS in patients with HCC (Figure 3). To further verify this result, the disease specific survival (DSS) of subgroups stratified according to GSDMs was analyzed. As a consequence, high expression of GSDME was correlated with poor DSS (Supplementary Figure 2). There was no significant difference between the other GSDMs and OS/DSS, despite their obviously up-regulated expression in HCC.

### Analysis of genetic alteration and homology in the GSDM family in patients with HCC

**Figure 4:**
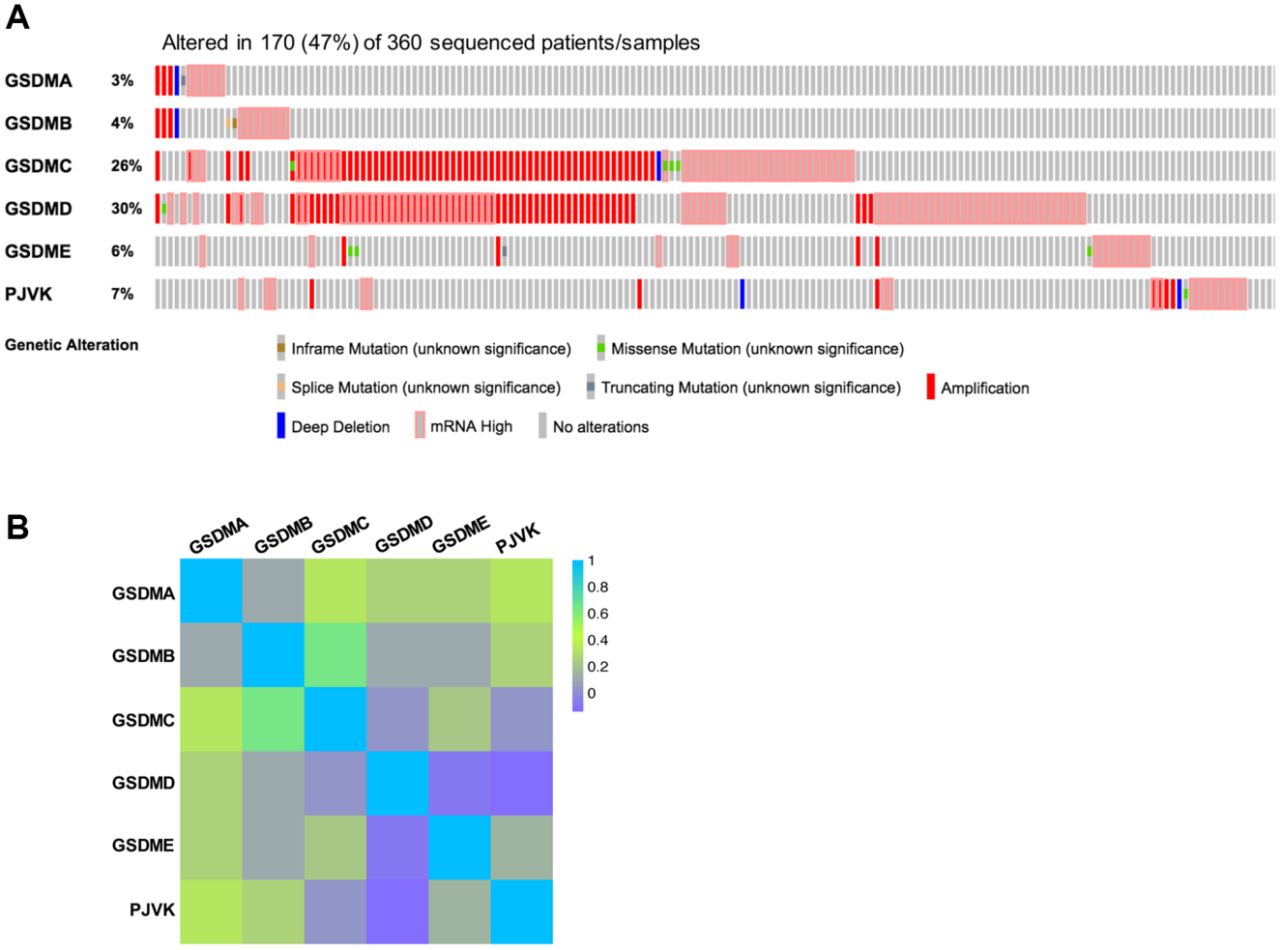
**Genetic alterations and correlation analysis of six GSDM family members in HCC.** (**A**) Genetic alteration profiles of six GSDM family members in HCC (cBioPortal). (**B**) Correlation between six GSDM family members in HCC by using GEPIA2.

The profiles of genetic alterations of each GSDM member were shown in Figure 4 with the application of the TCGA database and the cBioPortal online tool. On the whole, GSDM family genes are altered in 170 (47%) of 360 enrolled HCC patients. GSDMD had the highest genetic alteration rate (30%) among the GSDM family members, then the genetic alteration rate ranked from high to low as below: GSDMC (26%), PJVK (7%), GSDME (6%), GSDMB (4%) and GSDMA (3%) (Figure 4A). About the types of genetic alteration of GSDM members, amplification and mRNA high were the two main alteration types. The missense mutation, deep deletion, inframe mutation, and splice mutation happened rarely in GSDMs. Next, the homologous analysis inside the GSDM family members was onset, giving the result that GSDMC had a relatively strong correlation with GSDMB (R = 0.64) and GSDMA (R = 0.34), PJVK was found to be more similar with GSDMA (R = 0.34) (Figure 4B).

### Screening of GSDMs associated molecules and functional enrichment analysis

**Figure 5:**
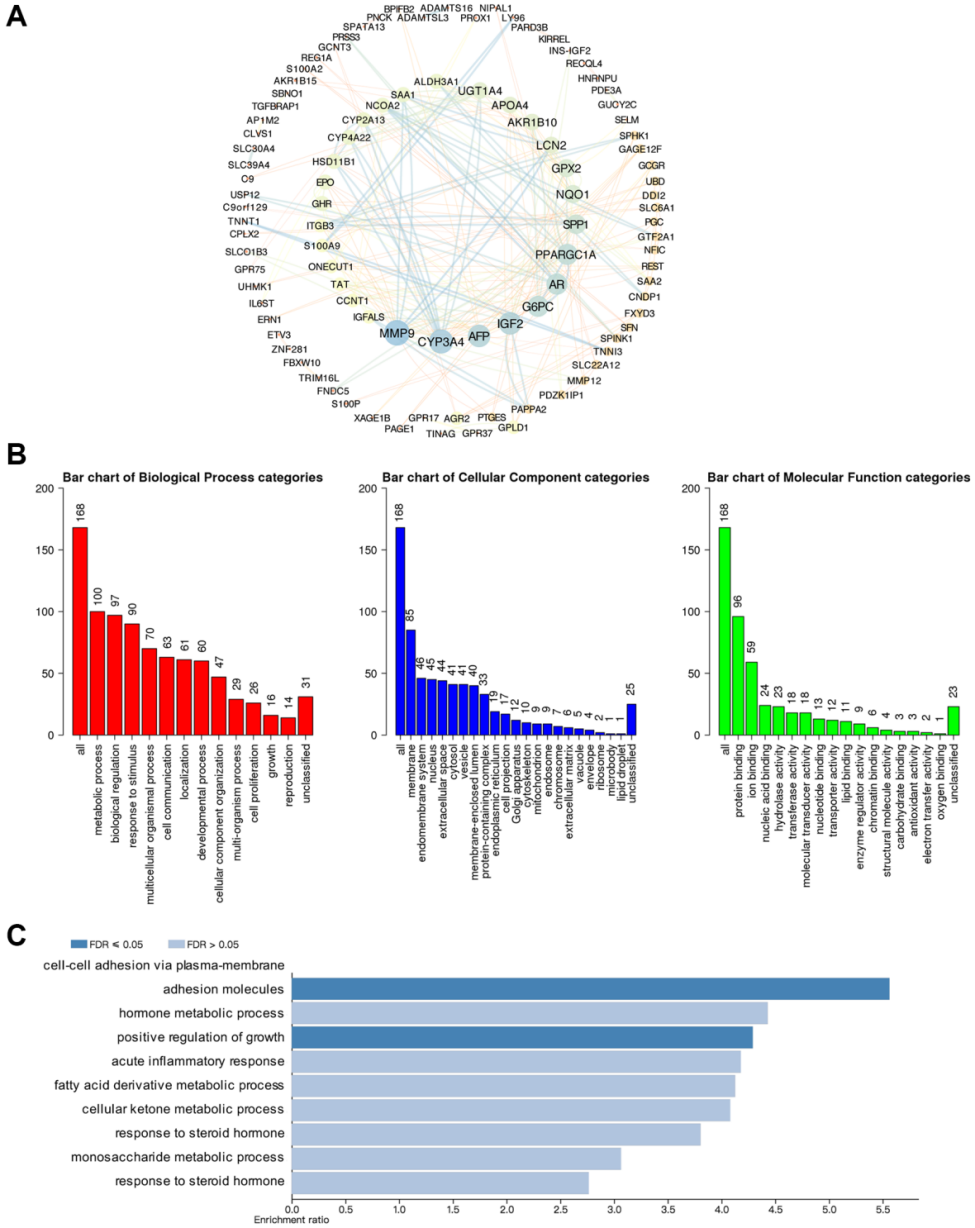
**Predicted functions and pathways of GSDMs and GSDM-associated co-expressed molecules in HCC.** (**A**) 168 GSDM-associated co-expressed molecules which were most frequently altered in HCC were identified by using cBioPortal, and protein-protein interaction (PPI) network was then conducted by Cytoscape. (**B**) Gene Ontology (GO) functional enrichment analysis of GSDM-associated co-expressed molecules was conducted by WebGestalt. (**C**) Kyoto Encyclopedia of Genes and Genome (KEGG) pathway analysis of GSDM-associated co-expressed molecules was conducted by WebGestalt.

cBioPortal and Cytoscape were used to screen out the top 168 genes which were co-expressed and associated with the GSDMs, then the protein-protein interaction (PPI) network was built base on it. Consequently, molecules like MMP9, CYP3A4, AFP, IGF2, G6PC, AR, and PPARGC1A had the highest possibility to cooperate with GSDMs, thus contributed to the initiation and progression of HCC (Figure 5A). To better understanding the associated function of GSDMs in HCC, the functional enrichment analysis was performed based on the 168 GSDM-correlated genes by using Gene Ontology (GO) annotation from the WebGestalt database. As presented in Figure 5B, the top-ranked biological processes regarding GSDMs were metabolic process, biological regulation, response to stimulus, multicellular organismal process, and cell communication. Moreover, the most highly enriched cellular components associated with GSDMs were membrane, endomembrane system, nucleus, and extracellular space. As for the molecular functions related to GSDMs, the protein binding, ion binding, nucleic acid binding, and hydrolase activity appeared on the list (Figure 5B). Furthermore, the Kyoto Encyclopedia of Genes and Genomes (KEGG) pathway analysis demonstrated nine potential signal pathways which were most likely related to GSDMs-associated molecules. The top three pathways were the cell adhesion pathway, growth regulation pathway and pathway accounted for hormone metabolic process (Figure 5C).

### The correlation of immune cell infiltration with GSDMs expression

**Figure 6:**
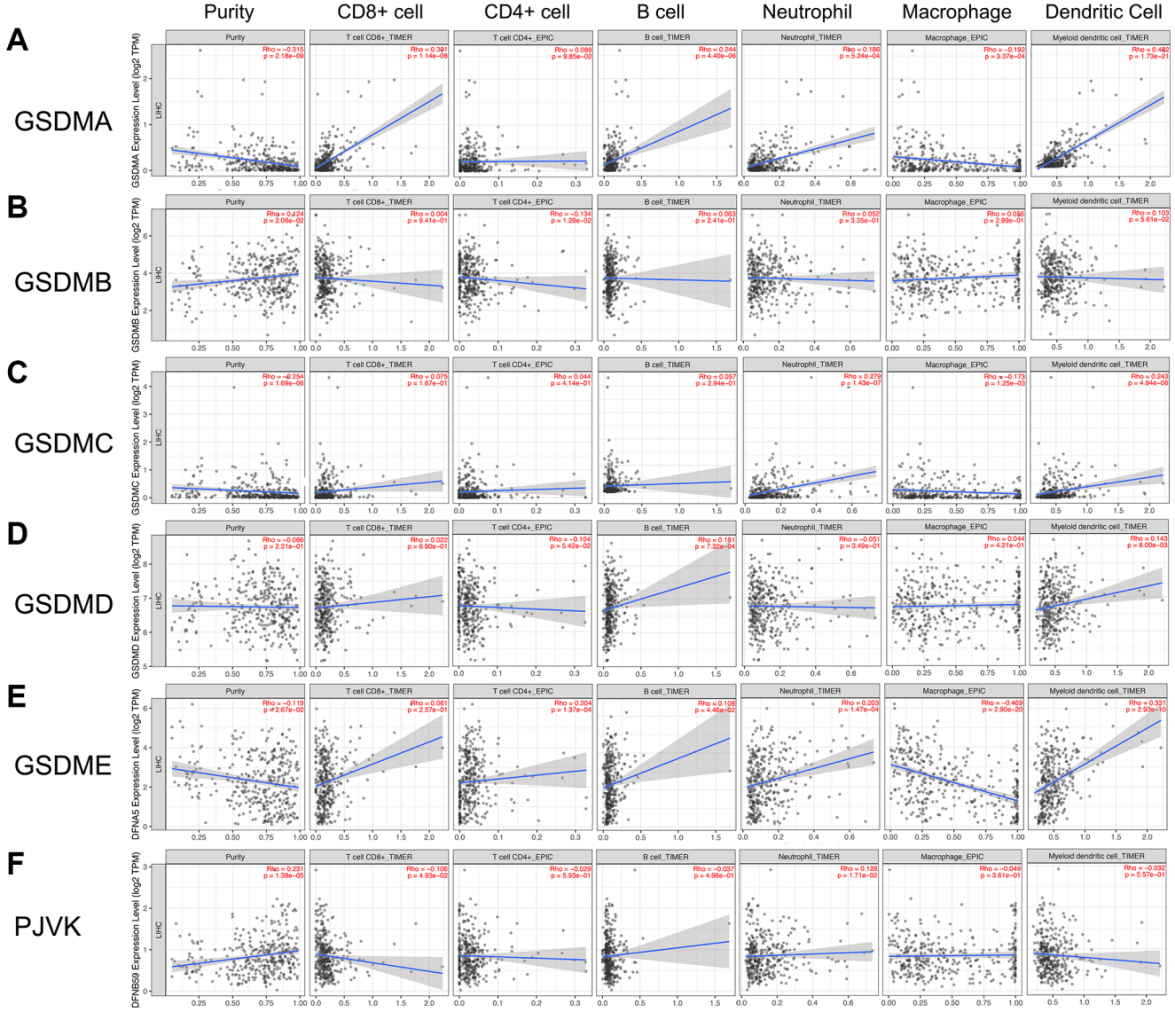
**Association of GSDMs mRNA expression levels with immune cell infiltration.** The effects of (**A**) GSDMA, (**B**) GSDMB, (**C**) GSDMC, (**D**) GSDMD, (**E**) GSDME, and (**F**) PJVK on the immune cell infiltration were evaluated by the TIMER2.0 database.

The various types of immune cells which infiltrated around tumor tissue were essential components of the tumor microenvironment [16]. Studies have shown that tumor biology behaviors including tumorigenesis, progression, and metastasis could be significantly affected by infiltrated immune cells [17, 18]. To explore if there was any relationship between GSDMs and immune cell infiltration, the TIMER 2.0 database was utilized in this section. As the results, GSDMA was positively correlated with infiltration of CD8+ T cell (*Rho* = 0.301, *P* = 1.14e-08), B cell (*Rho* = 0.244, *P* = 4.40e-06), neutrophil (*Rho* = 0.186, *P* = 5.24e-04), and dendritic cell (*Rho* = 0.482, *P* = 1.73e-21), while was negatively correlated with infiltration of macrophage (*Rho* = -0.192, *P* = 3.37e-04). GSDMB was only negatively correlated with CD4+ T cell infiltration (*Rho* = -0.134, *P* = 1.26e-02). GSDMD was only positively correlated with infiltration of B cell (*Rho* = 0.181, *P* = 7.32e-04) and dendritic cell (*Rho* = 0.143, *P* = 8.00e-03). The profiles of immune cell infiltration in the cases of GSDMC and GSDME exhibited a certain degree of similarity with that in GSDMA. PJVK was found to have negative correlation with CD8+ T cell infiltration (*Rho* = -0.106, *P* = 4.93e-02) and positively correlation with neutrophil (*Rho* = 0.128, *P* = 1.71e-02) (Figure 6).

### DNA methylation levels of the GSDMs in HCC patients

DNA methylation is a crucial part of the post-transcriptional modification that can negatively regulate gene expression, thereby get involved in the initiation and progression of cancer [19, 20]. We utilized the DiseaseMeth database here to further investigated the DNA methylation level of each GSDM family member in HCC tissues and made the comparison with that in normal liver tissues. As presented in Supplementary Figure 3, all of the six GSDM members showed remarkably decreased methylation levels in HCC tissues, suggesting that DNA methylation might be the partial mechanism underneath that made the GSDMs mRNA expression increased. Further mechanistic investigation of GSDMs methylation may bring benefits for the treatment of HCC patients.

## DISCUSSION

Pyroptosis is a recently identified form of programmed cell death that is mediated by GSDM family members [5, 21]. A variety of inflammation and immune responses happen concomitantly alongside the pyroptosis [22]. Increasing evidence these days indicated the indispensable role of GSDMs and pyroptosis in various cancer, making GSDM-mediated pyroptosis a novel and prospective research direction [23, 24]. For example, the caspase-3/GSDME axis could activate pyroptosis through the ROS/JNK pathway in breast cancer [25]. Moreover, it was reported that GSDMC inhibited the TGFβR2 activation, thereby acted as an oncogene and promoted the proliferation of colorectal cancer cells [14]. The above researches indicated that each GSDMs member has its particular functions in different cancer types, the same GSDMs molecule can either act as an oncogene or tumor-suppressive gene based on the tumor heterogeneity.

As far as our concern, there is no systematic analysis aiming at the role of GSDM family members in HCC. Hence in the present study, we first explored the differential mRNA expression of each GSDM family member in HCC tissues. We found that all the mRNA expressions of the six GSDM members were significantly increased in HCC tissues, suggesting their potential to act as oncogenes. This high consistency of expression tendency of the GSDM family highlighted the potential role of GSDM-associated pathway and function (such as pyroptosis) in HCC. However, in terms of the protein expression level, only GSDMB, GSDMD, and GSDME gave accordant results, which were highly expressed in HCC tissues.

Then the prognostic assessment based on the expression of GSDMs was performed to further test if GSDMB, GSDMD, and GSDME are competent to be oncogenes. Only GSDME was proved to have prognostic value for HCC patients because the high GSDME group had shorter OS and DSS. These data collectively draw our interest in the GSDME and made GSDME a potential biomarker that could predict the clinical outcome of HCC, as well as a therapeutic target for drug research and development. To our knowledge, there were many studies that echo our findings. For instance, GSDME was reported to be activated by miltirone, thereby induced pyroptosis and inhibited the tumor growth in HCC [26]. Furthermore, evidence proved that cisplatin-induced a high degree of pyroptosis in lung cancer cells through the caspase-3/GSDME pathway [27]. In another published paper, GSDME knockout in colorectal mice model showed decreased pyroptosis degree, attenuated tumor size, and number. Such antitumor effect was proved to be achieved by ERK1/2-dependent releasing of HMGB1 [28]. Therefore, we believed that GSDME had the potential to become a novel biomarker and therapeutic target on the premise of firm evidence of experiments and a clear illustration of the mechanism.

Our study is the first to show that GSDMs members shared the high expression profiles from tumor stage 1 to stage 3 in HCC, however, their expressions decreased to the normal levels for the HCC patients at stage 4. We speculated that although the GSDMs were involved in the progression of HCC, the poor general situation and decompensated status in the end stage of HCC might interfere with the increased expression of GSDMs.

Then the molecules such as MMP9 and AR were filtered as the co-expressed genes which were mostly related to GSDMs through the interactive network analysis. In addition, functional enrichment analysis in HCC revealed that the cell adhesion and the hormone metabolic process were the most relevant pathway in which GSDMs were involved. Cell adhesion is a key factor that participates in multi-steps of tumor progression such as tumor invasion, metastasis, and epithelial-mesenchymal transition [29]. HCC cells adhesion was reported to promote metastasis via the SMAD3 pathway in an exosome-dependent way [30]. Numerous kinds of hormone metabolisms are taken place in the liver — the hotbed that breeds the HCC. Therefore, the tumorigenesis and progression of HCC are indispensably influenced by hormones including AR. Recently an article uncovered that the mechanism of olaparib and enzalutamide in suppressing HCC progression is partially owing to the AR-mediated BRCA1 signaling pathway [31, 32]. Another study also demonstrated that AR dramatically reduced the HCC invasion and migration by targeting the miR-325/ACP5 pathway [33]. By king both our findings and published studies mentioned above into account, we believe further investigations along the direction of GSDMs, adhesion, and hormone metabolism were of great expectation.

Immune cell infiltration has been recognized these days as a pivotal parameter that is closely correlated with the progression and recurrence of tumors, the clinical outcome, and the efficacy of immunotherapy [34, 35]. Besides, infiltrated immune cells such as macrophage and CD8+T cells can be activated by GSDMs-mediated pyroptosis, thereby promote phagocytosis and anti-tumor immunity [15]. We found that there was a specific profile of immune cell infiltration according to the GSDMs in HCC. The majority of GSDMs members were positively correlated with the infiltration of B cells, neutrophils, and dendritic cells, however negatively correlated with macrophage. These data suggested that GSDMs-mediated pyroptosis might play crucial roles in anti-tumor immunity by affecting the profiles of immune cell infiltration.

As a key component of post-transcriptional modification, DNA methylation has been intensively reported to be involved in the regulation of cancer-associated genes [36, 37]. In our study, we found that the methylation level of all the GSDMs members is significantly reduced in HCC tissues, which puts us in mind of the increased expression of GSDMs in HCC tissues. These data highlighted the importance of DNA methylation in the GSDM family and clarified the direction of mechanism research towards DNA methylation was correct and worthy.

## CONCLUSIONS

In summary, the expression and function profiles of the GSDM family were disordered in HCC. The GSDM family (especially GSDME) had the potential to become essential biomarkers to better improve the diagnosis and prognosis of HCC, as well as provided insight for the development of therapeutic targets.

## MATERIALS AND METHODS

### UALCAN

The UALCAN database is a comprehensive and interactive web tool used for analyzing cancer data from OMICS (including TCGA, MET500, and CPTAC databases) [38]. UALCAN database was applied in this study to investigate the mRNA expression levels of GSDM family members in HCC. The mRNA expression of the GSDM members based on the HCC stage and HCC grade was further analyzed through UALCAN. In addition, the prognosis value of HCC patients based on GSDM expression was also analyzed using this database. The *p*-value equals to 0.05 was set as the cutoff with statistical significance. The databases used in the present study were summarized in Supplementary Table 1.

### The human protein atlas

The Human Protein Atlas is an online database that contains human proteins data derived from cells, tissues, and organs [39]. We employed this database to extract the immunohistochemistry results of each GSDM family member in HCC and normal liver tissues.

### Kaplan–Meier plotter

Kaplan–Meier Plotter is a public database that allows users to analyze the prognostic role of a mass of genes on survival in various types of cancer [40–42]. Here we used Kaplan–Meier Plotter to explore the correlation with GSDM family members of the overall survival and disease specific survival in patients with HCC. The *p*-value equals to 0.05 was set as the cutoff with statistical significance.

### cBioPortal

cBioPortal is a public web application designed for better utilization of genomics and clinical data in cancer researches [43, 44]. We use cBioPortal to retrieve a dataset containing 360 patients with HCC (TCGA, Firehose Legacy), then the co-expression and gene alteration analysis of GSDM family members were performed through cBioPortal.

### Cytoscape and WebGestalt

We used Cytoscape application to process the data of 168 co-expression genes of GSDMs which were obtained from the cBioPortal database (these gene names could be found in Supplementary Table 2) [45]. WebGestalt is a web tool for functional enrichment analysis based on gene sets in various biological contexts [46]. WebGestalt is adopted in this study to conduct the Gene Ontology (GO) analysis and the Kyoto Encyclopedia of Genes and Genomes (KEGG) analysis.

### TIMER 2.0

TIMER 2.0 is an open-access tool that allows users to make an assessment of immune cell infiltration based on the expression of specific genes in 32 types of cancer [47]. The module named “immune association” in this web tool was adopted to acquire the scatterplots that illustrate the correlation of GSDMs with different types of infiltrated immune cells (dendritic cells, macrophages, CD4+ T cells, CD8+ T cells, B cells, and neutrophils).

### DiseaseMeth2.0

DiseaseMeth2.0 is a professional online resource offering DNA methylation information in a variety of human diseases including cancer [48, 49]. The methylation status of GSDM family members in HCC and normal liver tissues were retrieved from this public resource. The *p*-value equals to 0.05 was set as the cutoff with statistical significance.

### Ethical statement

The authors are accountable for all aspects of the work in ensuring that questions related to the accuracy or integrity of any part of the work are appropriately investigated and resolved. None of the data have been previously published or appeared in copyrighted form elsewhere, and no previously published or unpublished data were cited in this paper. No ethics approval was required for this bioinformatics article, as it did not involve patients or patient data.

## Supplementary Materials

Supplementary Figures

Supplementary Tables
