# Supplementary material for: Integrated analysis of expression, prognostic value and immune infiltration of GSDMs in hepatocellular carcinoma
Source: Aging (Albany NY). 2021 Nov 3;13(21):24117–35. doi: 10.18632/aging.203669 (PMC8610125; doi:10.18632/aging.203669)
Supplement: Supplementary Figures [file aging-13-203669-s001.pdf]

SUPPLEMENTARY FIGURES

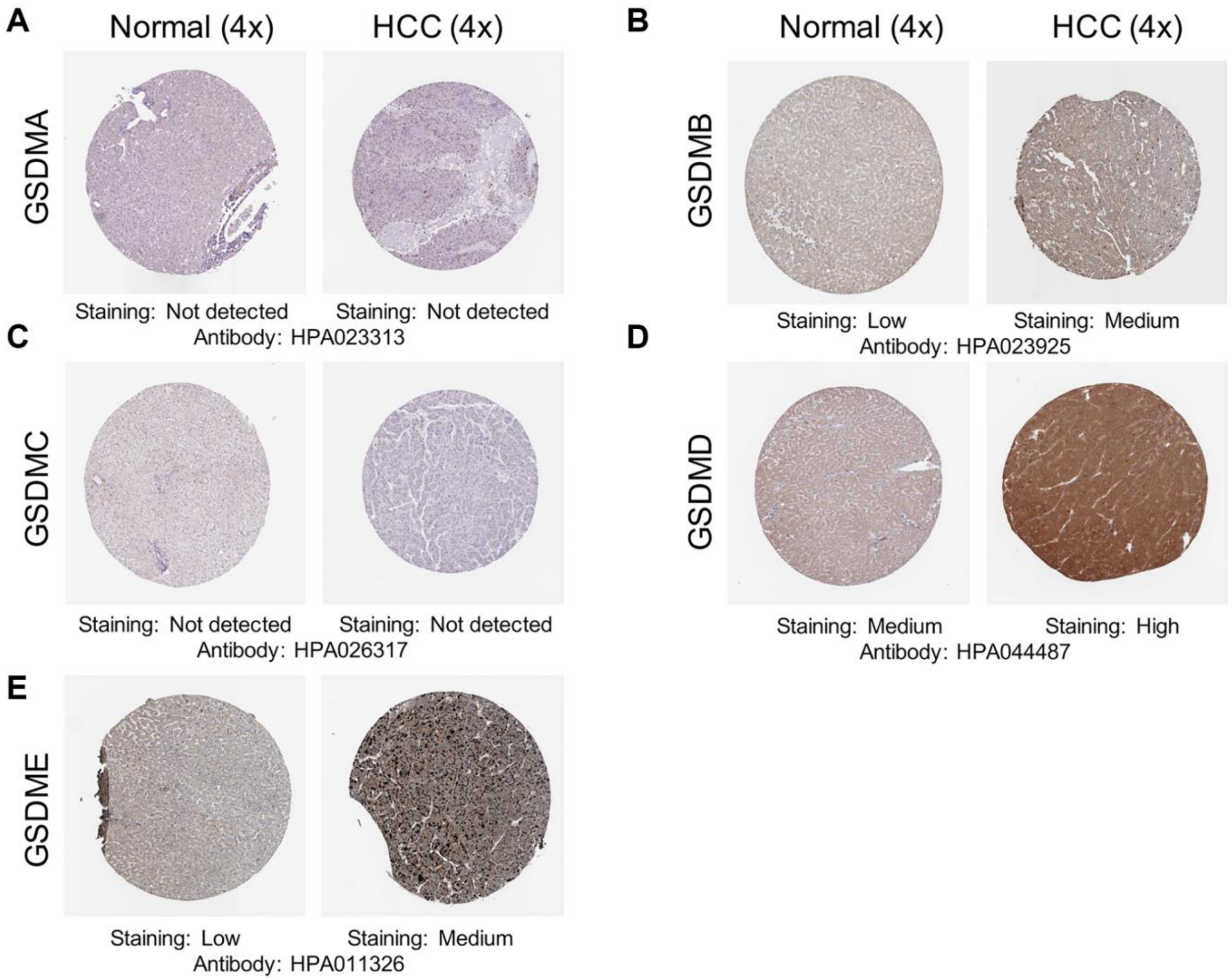

Supplementary Figure 1. Representative immunohistochemistry images of different GSDM family members between HCC tissues and normal liver tissues. (**A**–**E**) GSDMs protein expression data were retrieved from the THPA database (the Human Protein Atlas).

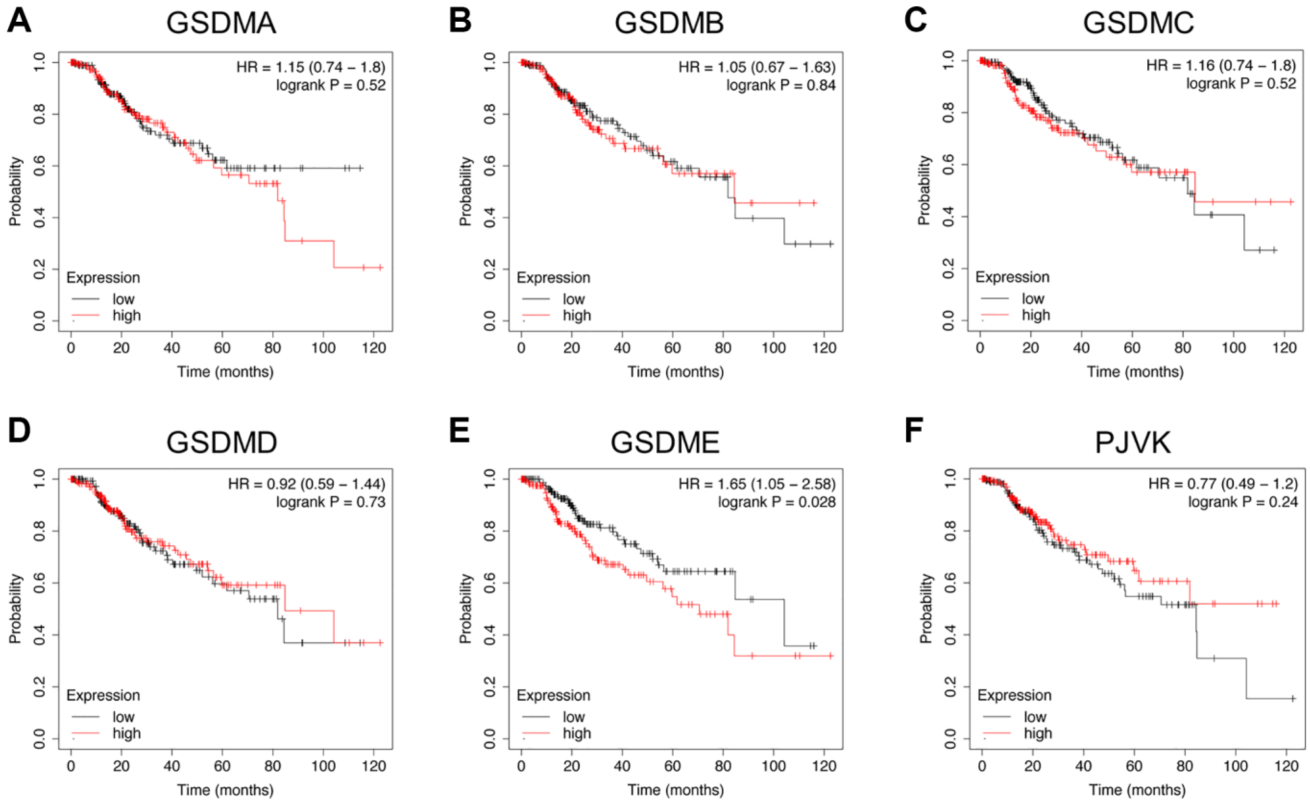

Supplementary Figure 2. Relationships between GSDMs mRNA expression levels and disease specific survival (DSS) of HCC patients (Kaplan-Meier plotter). (**A**–**F**) The disease specific survival (DSS) curve of six GSDM family members in patients with HCC. *P* < 0.05 was considered as the cutoff with statistical significance.

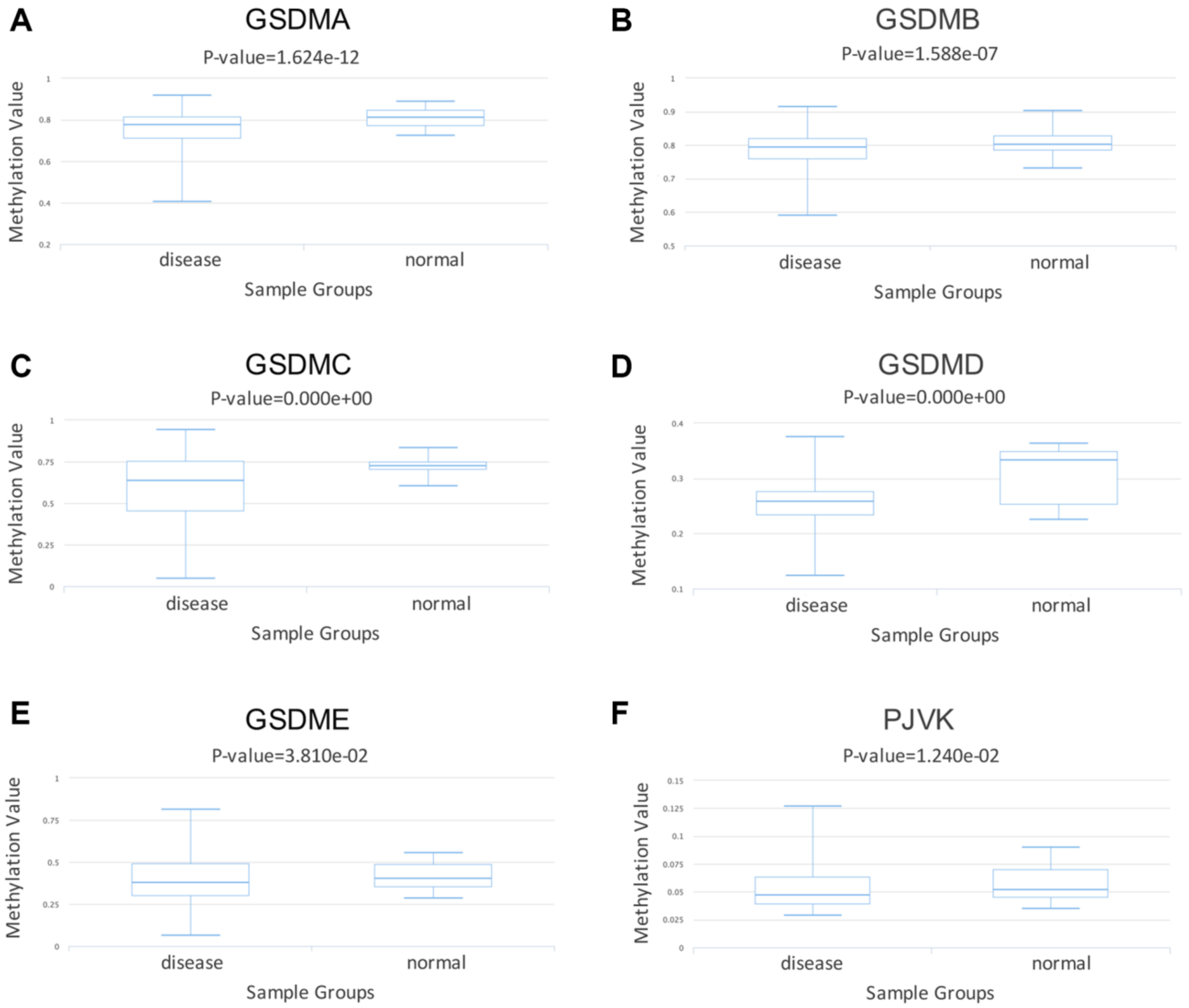

Supplementary Figure 3. DNA methylation levels of six GSDM family members in HCC. (**A**–**F**) The DNA methylation values of each GSDM family member between HCC tissues and normal liver tissues were evaluated by using DiseaseMeth. $P < 0.05$ was considered as the cutoff with statistical significance.
