# Supplementary material for: Integrated analysis of expression, prognostic value and immune infiltration of GSDMs in hepatocellular carcinoma
Source: Aging (Albany NY). 2021 Nov 3;13(21):24117–35. doi: 10.18632/aging.203669 (PMC8610125; doi:10.18632/aging.203669)
Supplement: Supplementary Tables [file aging-13-203669-s002.pdf]

SUPPLEMENTARY TABLES

Supplementary Table 1. Summary of bioinformatics database for analyzing the role of the GSDM family in HCC in this study.

Databases	Authors	Samples	Homepage links
UALCAN	Chandrashekar DS	Tissues	http://ualcan.path.uab.edu/index.html
GEPIA2	Tang Z, et al.	Tissues	http://gepia.cancer-pku.cn
The Human Protein Atlas	Anna Asplund, et al.	Tissues	https://www.proteinatlas.org
Kaplan-Meier plotter	Gyorffy B, et al.	Tissues	http://kmplot.com/analysis
cBioPortal	Cerami E, et al.	Tissues	http://www.cbioportal.org
WebGestalt	Liao Y, et al.	–	http://webgestalt.org
Cytoscape	Doncheva NT, et al.	–	–
TIMER2.0	Li T, et al.	Tissues	https://cistrome.shinyapps.io/timer
DiseaseMeth 2.0	Xiong Y, et al.	Tissues	http://bio-bigdata.hrbmu.edu.cn/diseasemeth

Supplementary Table 2. The GSDM-associated co-expressed molecules in HCC.

Gene	Log Ratio	*p*-Value	expression
RECQL4	1.13	1.83E-15	Altered group
ZNF281	−1.24	1.94E-14	Unaltered group
PPTC7	−1.38	7.95E-14	Unaltered group
ERN1	−1.26	1.21E-13	Unaltered group
ZNF366	−1.21	4.04E-13	Unaltered group
NFIC	−1.1	5.82E-13	Unaltered group
CCNT1	−1.32	1.72E-12	Unaltered group
SPATA13	−1.32	3.08E-12	Unaltered group
ROBO3	1.07	4.49E-12	Altered group
PROX1	−1.32	6.01E-12	Unaltered group
UHMK1	−1.33	7.34E-12	Unaltered group
RAPGEF6	−1.17	1.57E-11	Unaltered group
GDF7	−1.22	1.66E-11	Unaltered group
SHPRH	−1.03	2.11E-11	Unaltered group
IL6ST	−1.6	2.24E-11	Unaltered group
GTF2A1	−1.15	2.27E-11	Unaltered group
HIPK3	−1.36	2.31E-11	Unaltered group
RALGAPA2	−1.19	2.41E-11	Unaltered group
HACD2	−1.39	2.97E-11	Unaltered group
PCDHGB7	−1.29	6.17E-11	Unaltered group
USP12	−1.13	6.50E-11	Unaltered group
ZKSCAN8	−1.34	8.05E-11	Unaltered group
HS3ST3B1	−1.28	9.82E-11	Unaltered group
DDI2	−1.41	1.19E-10	Unaltered group
PVT1	1.14	1.20E-10	Altered group
NBPF10	−1.31	1.55E-10	Unaltered group
PRR34-AS1	1.02	1.76E-10	Altered group
ANKRD36BP1	−1.23	1.93E-10	Unaltered group
KIAA0754	−1.22	2.02E-10	Unaltered group
SYNC	1.86	2.05E-10	Altered group
SERINC5	−1.11	2.12E-10	Unaltered group
REST	−1.19	2.28E-10	Unaltered group
ETV3	−1.08	2.41E-10	Unaltered group
ONECUT1	−1.16	2.46E-10	Unaltered group
FNIP2	−1.2	2.52E-10	Unaltered group
GPR75	−1.09	4.29E-10	Unaltered group
PCDHGA6	−1.05	4.76E-10	Unaltered group
GPR17	−1.05	5.62E-10	Unaltered group
ZDHHC20	−1.2	7.82E-10	Unaltered group
TNFRSF18	1.1	9.24E-10	Altered group
PCDHGA12	−1.27	1.26E-09	Unaltered group
CDKL5	−1.09	1.40E-09	Unaltered group
DBH	−1.2	1.61E-09	Unaltered group
NBPF9	−1.16	1.82E-09	Unaltered group

GHR	−1.11	1.96E-09	Unaltered group
TNFSF12-TNFSF13	1.36	2.19E-09	Altered group
SBNO1	−1.15	2.20E-09	Unaltered group
TGFBRAP1	−1.08	2.28E-09	Unaltered group
PCDHGA2	−1.48	2.30E-09	Unaltered group
PCDHGB6	−1.17	2.57E-09	Unaltered group
N4BP2	−1.11	2.97E-09	Unaltered group
SLC30A4	−1.06	4.50E-09	Unaltered group
ADAMTSL3	−1.23	4.54E-09	Unaltered group
ITGB3	−1.1	6.08E-09	Unaltered group
NIPAL1	−1.24	6.81E-09	Unaltered group
CLVS1	1.44	8.04E-09	Altered group
FAT4	−1.07	8.57E-09	Unaltered group
AOC4P	−1.19	1.13E-08	Unaltered group
LY6E	1.24	1.59E-08	Altered group
SELENOM	1.11	1.63E-08	Altered group
SUCNR1	−1.08	1.82E-08	Unaltered group
GOLIM4	−1.03	2.66E-08	Unaltered group
NFATC2	−1.25	3.98E-08	Unaltered group
S100A9	1.17	5.70E-08	Altered group
TNNT1	1.41	6.92E-08	Altered group
PARD3B	−1.02	7.46E-08	Unaltered group
LY96	1.01	1.37E-07	Altered group
DAB1	−1.12	1.51E-07	Unaltered group
PKIB	1.24	1.77E-07	Altered group
S100A2	1.04	1.96E-07	Altered group
AQP7P3	1.1	2.35E-07	Altered group
GOLGA6B	−1.24	2.69E-07	Unaltered group
SFN	1.55	2.70E-07	Altered group
MMP9	1.29	3.88E-07	Altered group
ADRA1A	−1.34	3.89E-07	Unaltered group
SYNE4	1.03	4.48E-07	Altered group
PPARGC1A	−1.17	4.61E-07	Unaltered group
UBD	1.1	5.23E-07	Altered group
GPX2	1.46	5.42E-07	Altered group
INS-IGF2	−1.84	7.91E-07	Unaltered group
PCDHGA3	−1.1	8.65E-07	Unaltered group
PCDHB8	−1.06	8.81E-07	Unaltered group
NCOA2	−1.05	9.29E-07	Unaltered group
PDE3A	−1.06	1.01E-06	Unaltered group
FBXW10	1.3	1.01E-06	Altered group
KIRREL1	−1.15	1.12E-06	Unaltered group
TNNI3	1.01	1.21E-06	Altered group
FAM66D	1.04	1.22E-06	Altered group
IGFALS	−1.4	1.41E-06	Unaltered group
SLC5A11	1.32	1.57E-06	Altered group
AP1M2	1.92	1.71E-06	Altered group
CRYAB	1.05	1.83E-06	Altered group
SLC6A1	−1.01	2.05E-06	Unaltered group
PCDHGB5	−1.15	2.16E-06	Unaltered group
TRIM16L	1.01	2.32E-06	Altered group
PCDHGB2	−1.06	3.82E-06	Unaltered group
WASHC2A	−1.13	4.44E-06	Unaltered group
NQO1	1.54	4.57E-06	Altered group
PPP1R14D	1.07	4.77E-06	Altered group
GGTLC2	1.07	5.89E-06	Altered group
PCDHGA4	−1.02	6.60E-06	Unaltered group
INHBC	−1.01	7.35E-06	Unaltered group
TRIM54	1.17	7.70E-06	Altered group
AKR1B10	1.93	8.43E-06	Altered group
AR	−1.19	9.37E-06	Unaltered group
SNORD116-4	−1.04	2.07E-05	Unaltered group
FNDC5	−1.37	2.12E-05	Unaltered group
SPP1	1.59	2.73E-05	Altered group
TINAG	1.57	2.90E-05	Altered group
GUCY2C	1.38	3.83E-05	Altered group
ABCA8	−1.13	4.06E-05	Unaltered group
AVPR1A	−1.26	4.39E-05	Unaltered group

TRNP1	1.01	4.98E-05	Altered group
FXYD3	1.32	5.65E-05	Altered group
GPR37	−1.07	5.79E-05	Unaltered group
GPLD1	−1.1	6.01E-05	Unaltered group
SPHK1	1.04	6.52E-05	Altered group
GCNT3	1.14	7.47E-05	Altered group
MMP12	1.13	7.79E-05	Altered group
AKR1B15	1.33	8.03E-05	Altered group
LMTK3	1.06	8.07E-05	Altered group
SLC39A4	1.06	8.38E-05	Altered group
TAT	−1.37	1.18E-04	Unaltered group
LCN2	1.4	1.23E-04	Altered group
EPO	1.19	1.27E-04	Altered group
POPDC3	1.04	1.46E-04	Altered group
GCGR	−1.49	1.65E-04	Unaltered group
TMEM92	1.3	1.82E-04	Altered group
PAPPA2	−1.09	1.89E-04	Unaltered group
PTGES	1.09	2.20E-04	Altered group
PNCK	1.1	3.30E-04	Altered group
PITX1	1.21	4.42E-04	Altered group
CLDN4	1.2	4.70E-04	Altered group
SAA1	1.39	5.80E-04	Altered group
HHIPL2	1.05	5.88E-04	Altered group
PAGE1	1.02	6.38E-04	Altered group
CYP2A13	−1.05	7.10E-04	Unaltered group
SPINK1	1.57	7.36E-04	Altered group
CNDP1	−1.2	7.85E-04	Unaltered group
CYP4A22	−1.01	8.54E-04	Unaltered group
AGR2	1.33	9.43E-04	Altered group
AFP	1.41	9.51E-04	Altered group
ADAMTS16	−1.02	1.03E-03	Unaltered group
HSD17B13	−1.4	1.06E-03	Unaltered group
CYP3A4	−1.54	1.07E-03	Unaltered group
GAGE4	1.02	1.23E-03	Altered group
UGT2A1	1.03	1.30E-03	Altered group
UGT1A4	−1.23	1.58E-03	Unaltered group
ALDH3A1	1.43	1.60E-03	Altered group
RPS4Y1	1.64	1.85E-03	Altered group
S100P	1.28	1.90E-03	Altered group
PDZK1IP1	1.18	2.05E-03	Altered group
C9	1.36	2.75E-03	Altered group
SAA2	1.24	3.13E-03	Altered group
XIST	−1.55	3.48E-03	Unaltered group
FAM133A	1.02	4.14E-03	Altered group
HSD11B1	−1.01	4.61E-03	Unaltered group
PRSS3	1.12	4.90E-03	Altered group
CPLX2	1.34	5.46E-03	Altered group
BPIFB2	1.03	9.51E-03	Altered group
SLC22A12	1.02	9.70E-03	Altered group
IGF2	−1.11	9.83E-03	Unaltered group
PGC	1.19	0.0125	Altered group
REG1A	1.14	0.0138	Altered group
APOA4	1.11	0.0169	Altered group
EIF1AY	1.01	0.0191	Altered group
SLCO1B3	−1.01	0.0228	Unaltered group
XAGE1B	1.12	0.0315	Altered group
